# Spatial distribution of cerebral microbleeds and FLAIR hyperintensities on follow-up MRI after radiotherapy for lower grade glioma

**DOI:** 10.1016/j.redii.2023.100033

**Published:** 2023-08-14

**Authors:** Justyna Kłos, Reina W. Kloet, Hiska L. van der Weide, Kelvin Ng Wei Siang, Peter F. Sinnige, Miranda C.A. Kramer, Rudi A.J.O. Dierckx, Ronald J.H. Borra, Anouk van der Hoorn

**Affiliations:** a Department of Nuclear Medicine and Molecular Imaging, University of Groningen, University Medical Center Groningen, Hanzeplein 1, Groningen 9713GZ, the Netherlands; b Department of Radiology, University of Groningen, University Medical Center Groningen, Hanzeplein 1, Groningen 9713GZ, the Netherlands; c Department of Radiation Oncology, University of Groningen, University Medical Center Groningen, Hanzeplein 1, Groningen 9713GZ, the Netherlands

**Keywords:** Radiotherapy, Radiation, Glioma, Cerebral microbleeds, FLAIR hyperintensities, MRI, Brain, Radiotherapy-induced brain damage, Spatial distribution, Microbleed Anatomical Rating Scale

## Abstract

**Background and purpose:**

Cerebral microbleeds (CMBs) and fluid-attenuated-inversion recovery (FLAIR) hyperintensities on brain MRI scans after radiotherapy (RT) are considered markers for microvascular damage and related cognitive changes. However, the spatial distribution using existing scoring systems as well as colocation of these imaging biomarkers remain unclear, hampering clinical interpretation. This study aims to elucidate the distribution and colocation of these markers in patients with lower grade glioma (LGG).

**Materials and methods:**

CMBs were spatially classified on retrospective 1.5 T susceptibility weighted MRI scans according to the existing Microbleed Anatomical Rating Scale (MARS) and were additionally scored for being located in hippocampus, amygdala, cortex, white matter (WM), grey matter (GM), WM/GM junction and for their spatial relation to FLAIR hyperintensities. Scoring was performed for whole, ipsilateral and contralateral cerebrum (with respect to tumour bulk).

**Results:**

Fifty-one scans were included of which 28 had at least one CMB. The majority of CMBs were localized in the lobar area and in deep and periventricular white matter (DPWM) - generally in WM. Only few CMBs were found in GM. In scans obtained up to 7 years after RT completion the majority of CMBs were not colocalized with FLAIR hyperintensities.

**Conclusion:**

CMBs and FLAIR hyperintensities appear to be separate imaging biomarkers for radiation therapy induced microvascular damage, as they are not colocalized in patients with LGG, especially not early on after completion of RT.

## Highlights

• The longer the time between the completion of RT and the scan, the more CMBs appear.
• The higher the dose, the more FLAIR hyperintensities are generally observed.
• Compared to CMBs, FLAIR hyperintensities appear early after completion of RT.
• CMBs and FLAIR hyperintensities do not generally spatially overlap.

## 1 Introduction

Radiotherapy-induced brain damage (RIBD) occurring after treatment for brain tumours with a relatively long survival, such as lower grade gliomas (LGG) [1], may result in long-term cognitive side effects [2]. Accurate detection, classification, quantification, and interpretation of RIBD on radiological images is currently of high interest because of efforts to improve the treatment outcomes and quality of life of these patients. Among others, cerebral microbleeds (CMBs) and fluid-attenuated inversion recovery (FLAIR) hyperintensities are imaging biomarkers that have been linked to RIBD and cognitive decline [3] and are based on MRI images that are part of the routine clinical assessment. Both markers are considered to represent (micro)vascular injury although their exact mechanisms likely differ and are still unclear [4,5]. While the ever-ongoing research into the underlying exact mechanisms of these two biomarkers is beyond the scope of the current manuscript, knowledge regarding expected spatial distribution of CMBs and FLAIR hyperintensities and the degree of colocation of both markers could readily aid clinical interpretation. However, the spatial distribution assessed using existing scoring systems as well as the degree of colocation of these imaging biomarkers remain unclear in patients with LGG, in turn hampering clinical effective use of these biomarkers, prompting further investigation.

Regarding CMBs distribution, an influence of higher RT dose delivered to the brain tissue on CMBs formation has been reported [6,7]. Furthermore, some studies reported predominance of CMBs in the lobar location [8,9]. However, which lobe is most affected seems to depend on the tumour location, and as a result is the same lobe which received the highest RT dose. For example, in case of head and neck cancer, CMBs occurred most often in the frontal and temporal lobe [10], while in case of medulloblastoma they occurred mostly in the occipital lobe [8]. Although some neurological conditions seem to have specific spatial patterns of CMBs [11], there are no studies specifically focused on the detailed spatial distribution of CMBs on MRI follow-up scans after treatment for LGG.

For FLAIR hyperintensities an association with RT also has been demonstrated, with studies indicating that the distribution of RIBD-related FLAIR hyperintensities depends on the type of RT, with the distribution being symmetrical after whole brain radiotherapy (WBRT) [12] and rather asymmetrical after focal RT [13]. When it comes to focal RT for low grade glioma, FLAIR hyperintensities seem to occur mainly in the areas of mid and high RT dose [14].

The interdependence between CMBs and FLAIR hyperintensities has shown contradicting results. One study found no topographic correlation between CMBs and FLAIR hyperintensities [15]. The other study which used 7 T MRI, which is still predominantly a research tool and rarely applied in clinical brain tumour workflows, found that CMBs occurred mainly within the areas of T2-hyperintense lesions, especially in the first 2.5 years since RT completion [16]. The percentage of CMBs occurring outside white matter (WM) hyperintensities became dominant only 3 years from the time of RT. Thus, the patterns of occurrence and the interdependence between CMB and FLAIR for use in current clinical practice are still unclear.

In this study, we aim to elucidate the spatial distribution and colocation of CMBs and FLAIR hyperintensity imaging biomarkers through providing an in-depth description of the spatial distribution of CMBs in the cerebrum of patients who underwent RT for a LGG and a routine 1.5 T follow-up MRI scan.

## 2 Methods

### 2.1 Study population

This retrospective study is based on medical records of adults who underwent RT for LGG (grade 1, 2 or 3 World Health Organisation (WHO)) and had a follow up MRI scan at the University Medical Centre Groningen (UMCG), Groningen, the Netherlands. Data were extracted in the context of a larger retrospective study concerning RIBD in patients with LGG. This study was approved by the institutional review board and the need for written informed consent was waived. To minimize the potential effects of other conditions on the brain tissue, patients were excluded if their medical history included hydrocephalus requiring neurosurgery, other than tumour-related brain disease, other serious conditions like other cancers, sepsis, lung embolism, serious heart disease, brain injury, or second line RT. Hypertension, diabetes, and epilepsy were not an exclusion criterium considering their common occurrence.

The most recent available follow-up MRI scan of each patient was used for the analysis, provided the scan was acquired on 1.5 T MRI with a protocol including SWI, isotropic MPRAGE T1-weighted and isotropic FLAIR-weighted imaging of sufficient quality for the analysis. RT dose information was extracted from the relevant radiotherapy systems for the whole, ipsilateral and contralateral cerebrum with the tumour bulk as a reference.

### 2.2 MRI acquisition parameters

MRI scans were acquired on 1.5 T Magnetom Aera (*N* = 30) and 1.5 T Magnetom AvantoFit (*N* = 21) scanners (Siemens Healthineers, Erlangen, Germany). Image acquisition parameters were: 1) T1: voxel size 0.98 × 0.98 × 1 mm, slice thickness 1 mm, repetition time (TR) 2200 ms, echo time (TE) 2.63–2.67 ms, inversion time (TI) 900 ms, flip angle 8°, field of view (FOV) (250–257)*250; 2) FLAIR: voxel size 1 × 1 × 1 mm, TR 5000 ms, TE 335 ms, TI 1800 ms, flip angle 120°, FOV (227–260)*260; 3) SWI: voxel size 0.72 × 0.72 × 2 mm, TR 49 ms, TE 40 ms, flip angle 15°, FOV (186–201)*230.

### 2.3 Image analysis

The cerebrum was assessed for CMBs count according to previously published definitions [11,17]. CMBs were marked as regions of interest (ROIs) on SWI images independently by two experienced observers (JK, RK) in a blinded fashion. In case of discrepancies, final consensus was achieved through the assessment of a third experienced observer (AvdH). The observers did not use clinical information during image analysis. Access to other MRI series within the same exam as well as to previous MRI scans (if available) was provided to the readers in order to improve the differentiation of CMBs from other potential visually similar changes on brain MRI.

**Fig. 1:**
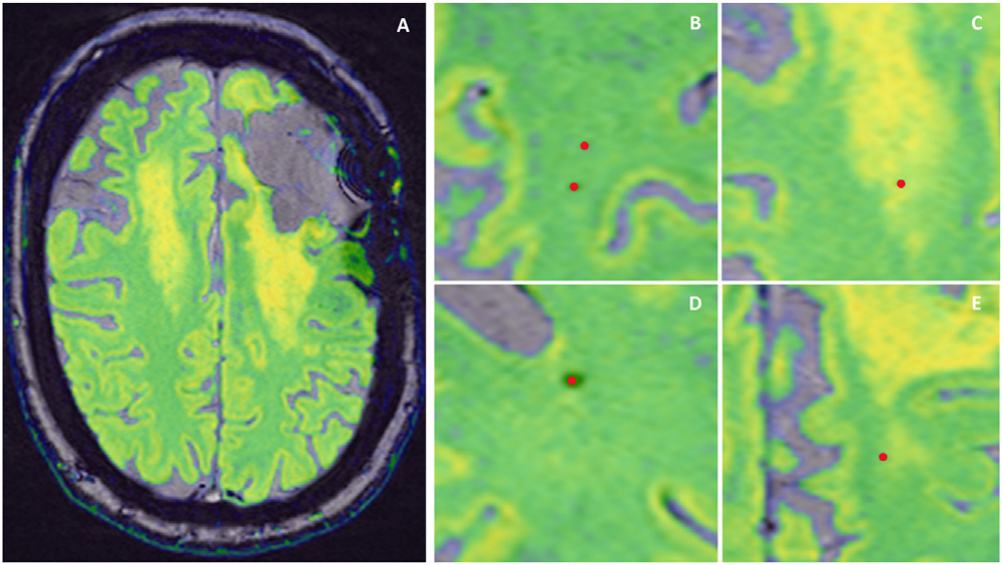
Methods used to assess the spatial distribution of CMB within FLAIR hyperintensities. A. Fused MRI SWI (grey, bottom) and FLAIR (green – yellow, top) images. Green colour showing the areas of normally appearing brain tissue and yellow colour showing FLAIR hyperintensities and grey matter. B. CMB (red dot) in normally appearing brain tissue. C. CMB within FLAIR hyperintensity. D. CMB within potential FLAIR hyperintensity (dispersed hyperintense voxels differing from normally appearing brain tissue, but not forming a cluster of hyperintensity. E. CMB on the border between normally appearing brain tissue and FLAIR hyperintensity.

To assess the overlap between CMBs and FLAIR hyperintensities, the SWI images with CMB ROIs were fused with FLAIR images in Horos v3.3.5 (Nimble Co LLC d/b/a Purview, Annapolis, MD USA, horosproject.org). The observers (JK and RK) independently classified and labeled the location of each CMB in relation to FLAIR hyperintensity as follows: (1) area of normal tissue, (2) area of obvious FLAIR hyperintensity, (3) border between FLAIR hyperintensity and normal tissue, (4) area of potentially developing FLAIR hyperintensity, defined as an area without specific clustered FLAIR hyperintensity but with many separate voxels of higher intensity than the surrounding area (see Fig. 1). The final consensus was achieved through the input of a third observer (AvdH). CMB count was provided for cerebrum: whole, ipsilateral and contralateral (with tumour bulk as a reference). For structural distribution, we divided CMBs into lobar and deep cerebrum based on the Microbleed Anatomical Rating Scale (MARS) [18]. Within the lobar distribution, we additionally specified the CMB count in the cortex, hippocampus and amygdala, as this distinction is not part of the MARS scale. For tissue distribution, we specified the number of CMBs in the WM, grey matter (GM) and GM/WM junction, in line with the Brain Observer MicroBleed Scale (BOMBS) [19].

Additionally, volumes of whole, ipsilateral and contralateral FLAIR hyperintensities in the cerebrum were assessed as a reference. The FLAIR hyperintensities were semi-automatically delineated through a combination of automated initial WM hyperintensity segmentation by cNeuro cMRI v1.11.0 (Combinostics Oy, Tampere, Finland, www.cneuro.com) and subsequent manual quality adjustments of the masks in ITK-SNAP v3.8.0 [20] (www.itksnap.org) – this because the intended use of cNeuro is for dementia and it is not intended to be used in the context of major anatomical brain changes, such as brain tumours. However, we found that in the context of this study cNeuro produced better than expected WM segmentations, despite the fact that in all scans both small and larger anatomical changes related to brain tumour (therapy) were present. Therefore, as such cNeuro greatly sped up the overall segmentation process of FLAIR hyperintensities.

### 2.4 Statistical analysis

Statistics were performed using IBM SPSS Statistics for Macintosh, Version 27.0, Released 2020, Armonk, NY, USA. Parametric or non-parametric tests were used, depending on the distribution normality of the tested variables. A two-sided P-value < 0.05 was defined as significant. Spearman Correlations were used to correlate the number of CMBs and the volume of FLAIR hyperintensities with time since RT completion, age at the time of RT, age at the time of the scan, and RT dose delivered to cerebrum. Related-Samples Wilcoxon Signed Rank Test was used to compare the number of CMBs and the volume of FLAIR hyperintensities between the ipsilateral and contralateral cerebrum. Paired Samples T-Test was used to compare the RT dose volume between the ipsilateral and contralateral cerebrum. To assess the potential confounding effects of chemotherapy, chemoradiotherapy, number of surgeries, hypertension, diabetes, and smoking, a Generalized Linear Estimation test was performed.

## 3 Results

In total, 51 patients were eligible for this study, 20 females and 31 males. The mean age at the time of RT and at the time of the MRI scan used in this study was 44 (22–79; SD =12.6) and 46 (23–79; SD = 12.5) years, respectively. The median time between RT end and the MRI scan was 20 months (IQR = 6–39). In most cases the tumour bulk was located supratentorial and in the frontal (45.1%) or temporal lobe (17,6%). In two cases tumour was located infratentorial, in one case in the ventricles, and in a single patient bilaterally in the frontal lobes – therefore, with no distinguishable ipsilateral or contralateral side. Further clinical characteristics are provided in Table 1.

**Table 1:** Clinical characteristics of patients.

Clinical characteristics	number (%)
Hypertension	3 (5.9)
Diabetes	4 (7.8)
Epilepsy	44 (86.3)
Smoking	24 (47.1)
**Tumour type**	**number (%)**
astrocytoma, WHO grade 1	1 (2.0)
astrocytoma IDH1 mutated, WHO grade 2	18 (35.3)
astrocytoma IDH1 wild type, WHO grade 2	3 (5.9)
astrocytoma IDH1 mutated, WHO grade 3	5 (9.8)
astrocytoma IDH1 wild type, WHO grade 3	3 (5.9)
oligodendroglioma, WHO grade 2	16 (31.4)
oligodendroglioma, WHO grade 3	3 (5.9)
subependymoma, WHO grade 1	1 (2.0)
ependymoma, WHO grade 2	1 (2.0)
**Tumour location**	**number (%)**
Frontal	23 (45.1)
Temporal	9 (17.6)
Insular	4 (7.8)
Other	15 (29.5)
**Chemotherapy**	**number (%)**
None	11 (21.6)
Chemoradiotherapy TMZ	2 (3.9)
Chemoradiotherapy TMZ + TMZ sequential to RT	4 (7.8)
PCV sequential to RT	29 (56.9)
PCV and TMZ sequential to RT	1 (2.0)
TMZ sequential to RT	4 (7.8)
**Surgery**	**number (%)**
Biopsy only	8 (15.7)
Single	31 (60.8)
Multiple	12 (23.5)
**RT type**	**number (%)**
3D CRT	3 (5.9)
VMAT+3D CRT	18 (35.3)
VMAT	16 (31.4)
IMRT	5 (9.8)
Fractionated SRT	4 (7.8)
IMPT	5 (9.8)
**RT dose [Gy]**	**mean (SD)**
cerebrum	47.90 (21.47)
IL cerebrum	30.63 (10.34)
CL cerebrum	17.55 (10.22)

WHO – World Health Organization; IDH1 – isocitrate dehydrogenase 1 mutation; RT – radiotherapy; TMZ – temozolomide; PCV – procarbazine, lomustine and vincristine; 3D CRT – three-dimensional conformal radiation therapy; VMAT – volumetric modulated arc therapy; IMRT – intensity-modulated radiation therapy; SRT – stereotactic radiotherapy; IMPT – intensity-modulated proton therapy; IL – ipsilateral; CL – contralateral.

Most patients underwent photon RT (90.2%) and only 5 (9.8%) underwent proton RT. The commonly used RT modalities were volumetric modulated arc therapy (VMAT) (31.4%) and VMAT in combination with three-dimensional conformal radiation therapy (3D CRT) (35.3%). The mean RT dose delivered to the ipsilateral hemisphere (*M* = 30.63 Gy, SD = 10.34) was significantly higher than the RT dose delivered to the contralateral hemisphere (*M* = 17.55 Gy, SD = 10.22) *t* = −10.47, df = 46, *P* < 0.001.

### 3.1 CMBs

**Fig. 2:**
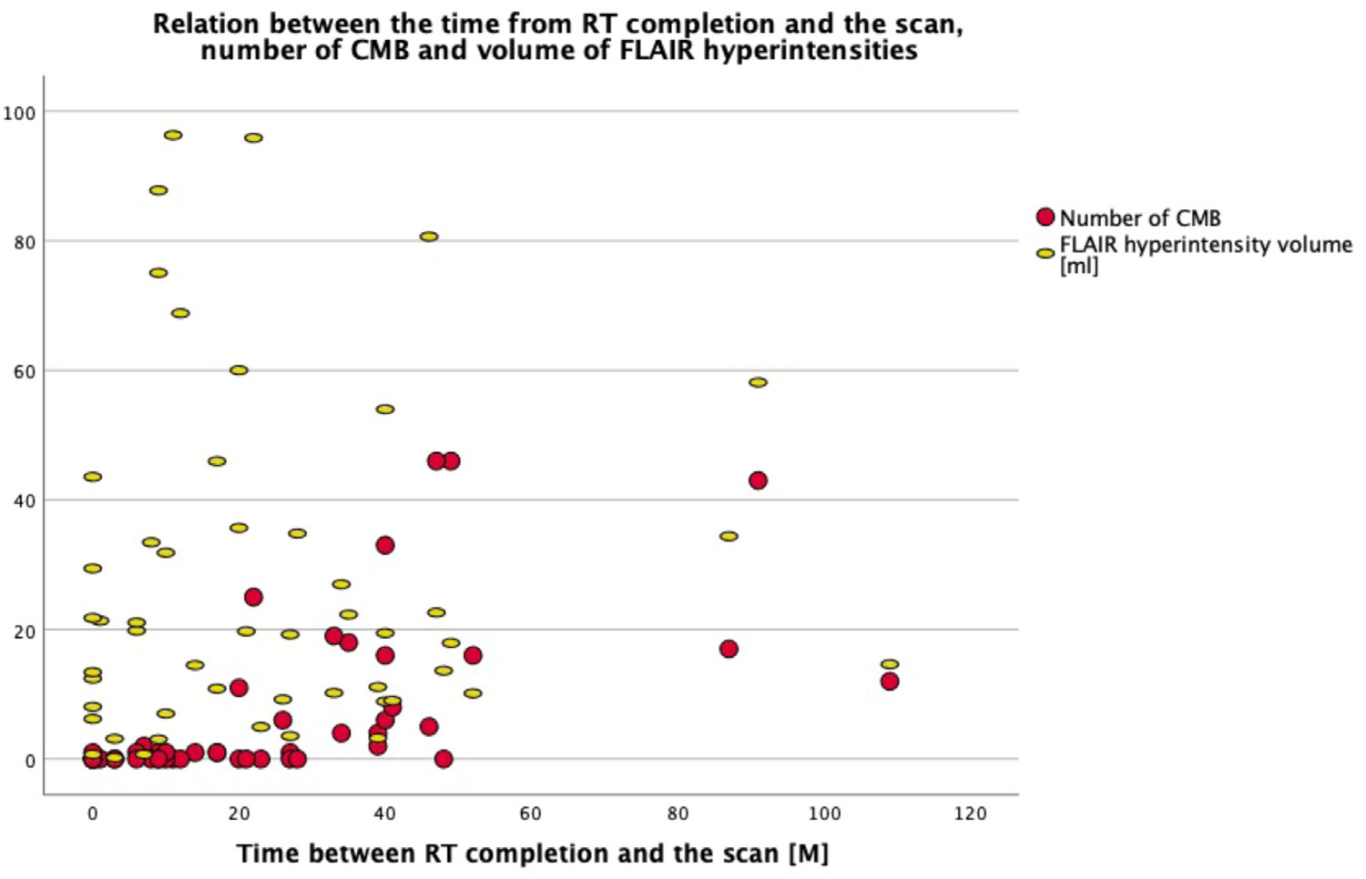
The number of CMBs and the volume of FLAIR hyperintensities (in millilitres) per scan (*N* = 51) on a timeline from completion of RT to the scan (in months). The number of CMB was strongly correlated with the time between the scan and completion of RT. Only a few CMBs were found on the scans acquired less than 20 months after RT completion but the number significantly increased on the scans at least 20 months from RT completion. The volume of FLAIR hyperintensities was not correlated with the time between RT completion and the scan.

The number of CMBs strongly correlated with the duration of the time-interval between completion of RT and the MRI scan (*N* = 51, *r* = 0.715, *P* < 0.001) (Fig. 2). In addition, the number of CMBs did not correlate with potential confounders such as age at the time of RT nor with age of the patient at the time of the scan. Furthermore, the number of CMBs was not statistically significantly correlated with chemotherapy, surgeries, hypertension, diabetes or smoking.

**Fig. 3:**
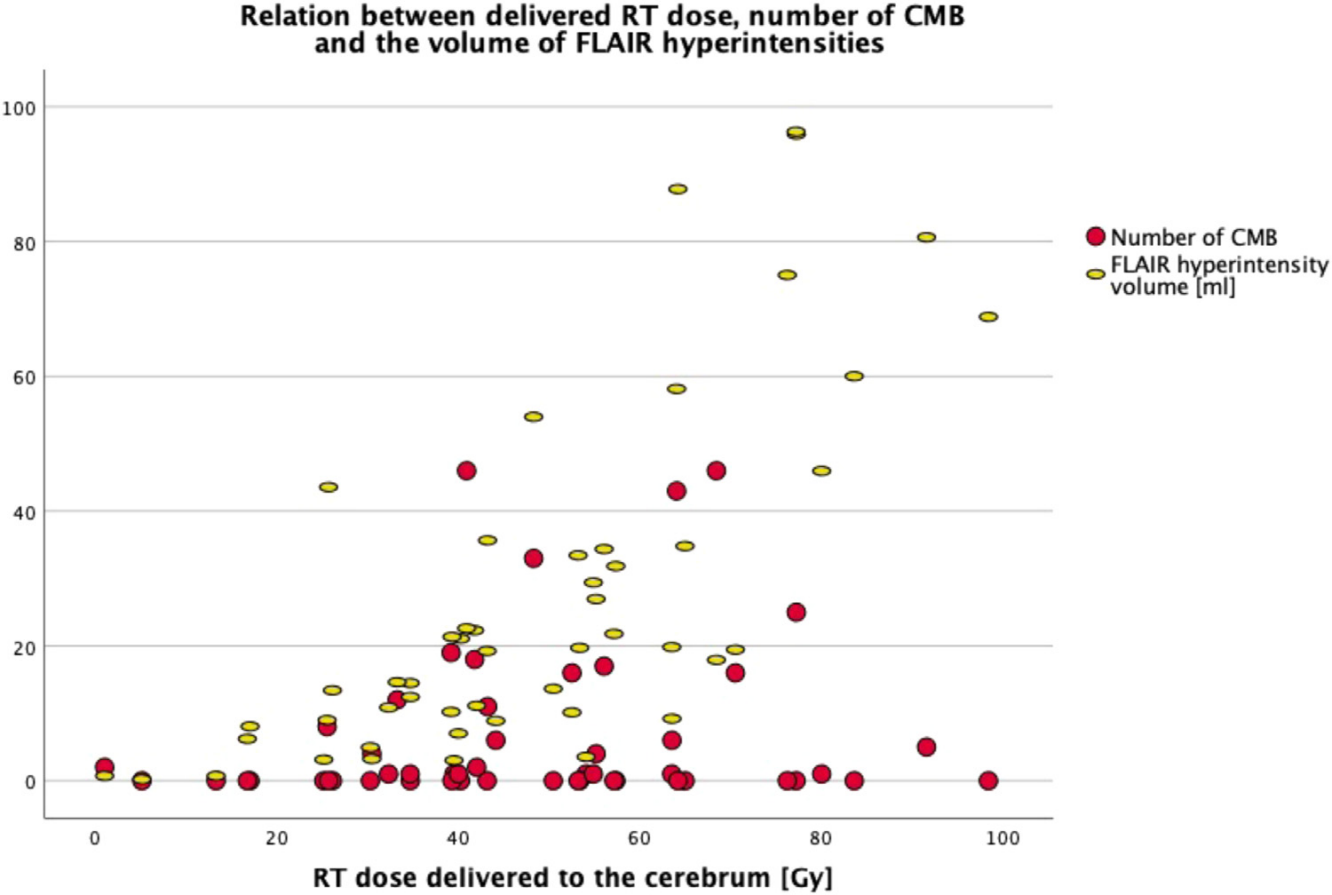
Relation between the RT dose, the number of CMB and the volume of FLAIR hyperintensities in the cerebrum (*N* = 51). The volume of FLAIR hyperintensities strongly correlates with the RT dose delivered to the cerebrum, but the number of CMB did not correlate with the RT dose delivered to the cerebrum.

Furthermore, correlations between the number of CMBs and the RT dose delivered to the cerebrum were non-significant, except for a weak correlation observed between the RT dose delivered to the contralateral cerebrum and the number of CMBs in the contralateral cerebrum (*n* = 47, *r* = 0.303, *p* = 0.039) (Fig. 3).

Within the study population, CMBs were present in 28 (54.9%) scans only, which all were obtained in patients receiving photon-based RT. For these scans, further assessment of the spatial distribution of CMBs was performed. The majority of the scans without any identified CMB were acquired within the first 20 months since RT completion (Fig. 2). The median number of CMBs in the cerebrum was 6.00 (IQR = 1.00–17.00). The number of CMBs in the ipsilateral cerebrum was not significantly higher than in the contralateral cerebrum (*Z* = 1.3, *p* = 0.195).

Regarding division of CMBs according to the MARS scale, the highest median number of CMBs occurred in the lobar location (median 5.50, IQR = 1.00–15.00), predominantly in the frontal and parietal lobe. In the additionally identified structures, there were three CMBs in the amygdala, two in the hippocampus and a single CMB in the cortex. Within deep cerebral locations (MARS scale), CMBs occurred mostly in deep and periventricular white matter (DPWM). Only five CMBs were found in the corpus callosum, a single CMB was found in the basal ganglia, thalamus and internal capsule, while none were found in the external capsule. The additional distribution of CMBs in accordance with tissue type was highest in the WM (median 5.00, IQR = 1.00–12.00), while only a few CMBs occurred in the GM.

**Fig. 4:**
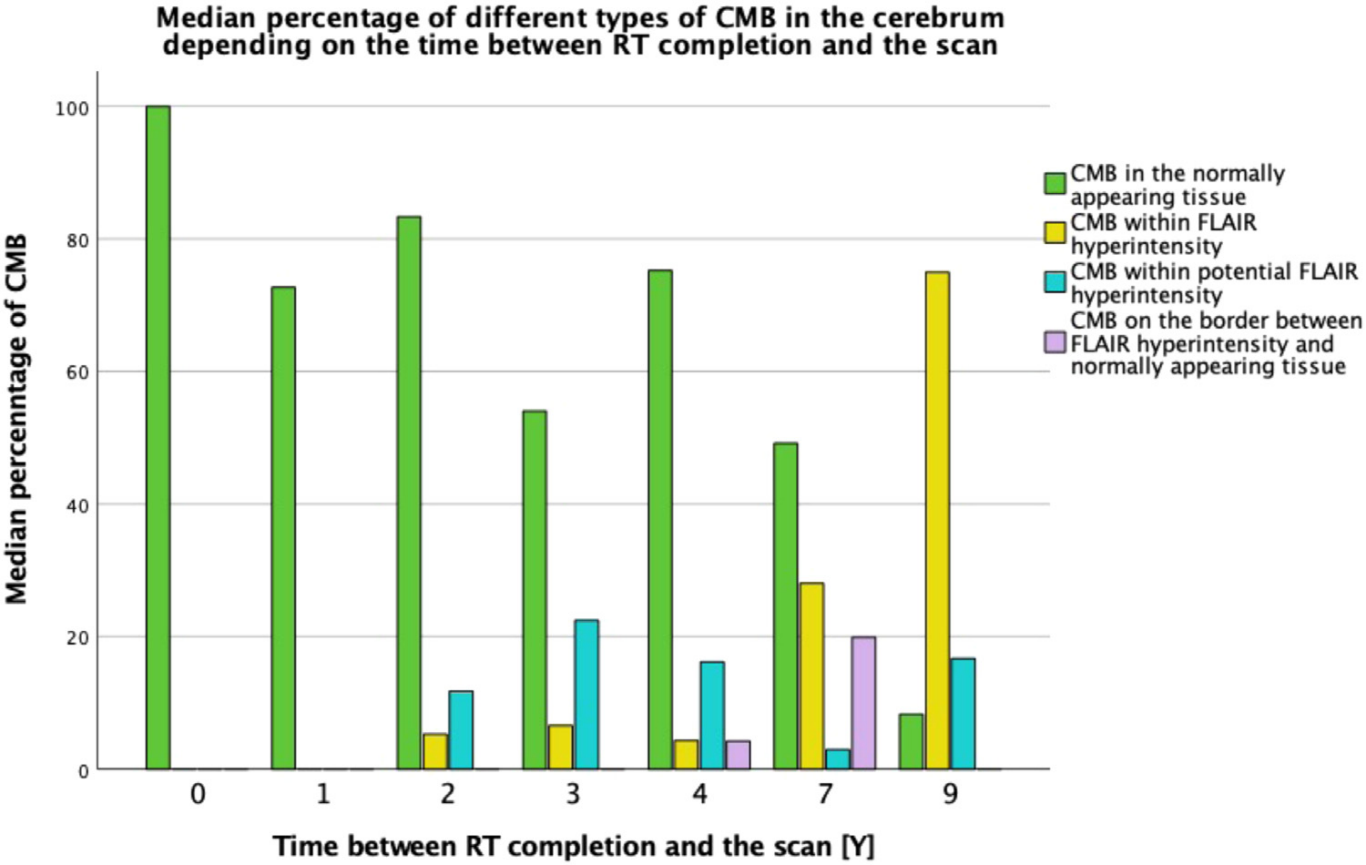
Median percentage of different types of CMB in cerebrum (*N*= 28) depending on the time between RT completion and the scan (in years). Within the first 7 years, CMBs predominantly occurred in the normally appearing cerebral tissue on FLAIR images. Only 9 years after completion of RT CMBs occurred predominantly within FLAIR hyperintensities.

Regarding the overlap between CMBs and FLAIR hyperintensities, the highest median number of CMBs occurred in the areas of normally appearing cerebral tissue on FLAIR images (median 3.50, IQR = 1.00–13.75). Detailed results including ipsilateral and contralateral side are available in Table 2. Furthermore, the median percentage of CMBs in the normally appearing cerebral tissue was highest up to 7 years from completion of RT, in whole, ipsilateral and contralateral cerebrum. Only 9 years after completion of RT the median percentage of CMBs occurring within FLAIR hyperintensity was higher, specifically because of an increase in observed CMBs occurring within FLAIR hyperintensities in the ipsilateral cerebrum (Fig. 4 and Supplementary Fig. 1).

**Table 2:** Spatial distribution of CMBs and volume of FLAIR hyperintensities.

		Whole cerebrum *N* = 28			Ipsilateral cerebrum *N* = 25			Contralateral cerebrum *N* = 25		
**CMB distribution**		**median**	**range**	**IQR**	**median**	**range**	**IQR**	**median**	**range**	**IQR**
		6.00	1–46	1.00– 17.00	6.00	0–28	1.00–12.50	1.00	0–27	0.00–8.50
**CMB distribution according to MARS**		**median**	**range**	**IQR**	**median**	**range**	**IQR**	**median**	**range**	**IQR**
Lobar		5.50	0–38	1.00–15.00	6.00	0–19	1.00–11.50	1.00	0–23	0.00–7.00
	Frontal	1.00	0–28	0.00–7.75	0.00	0–13	0.00–5.00	0.00	0–20	0.00–5.00
	Parietal	1.00	0–14	0.00–4.75	1.00	0–10	0.00–4.00	0.00	0–6	0.00–1.00
	Temporal	0.00	0–11	0.00–3.50	0.00	0–7	0.00–2.00	0.00	0–7	0.00–0.00
	Insular	0.00	0–2	0.00–0.00	0.00	0–2	0.00–0.00	0.00	0–1	0.00–0.00
	Occipital	0.00	0–10	0.00–1.00	0.00	0–3	0.00–0.00	0.00	0–7	0.00–0.50
	Cortex*	0.00	0–1	0.00–0.00	0.00	0	0.00–0.00	0.00	0	0.00–0.00
	Hippocampus*	0.00	0–1	0.00–0.00	0.00	0–1	0.00–0.00	0.00	0–1	0.00–0.00
	Amygdala*	0.00	0–1	0.00–0.00	0.00	0–1	0.00–0.00	0.00	0	0.00–0.00
Deep		0.00	0–14	0.00–2.00	0.00	0–10	0.00–1.00	0.00	0–5	0.00–1.00
	Basal ganglia	0.00	0–1	0.00–0.00	0.00	0	0.00–0.00	0.00	0–1	0.00–0.00
	Thalamus	0.00	0–1	0.00–0.00	0.00	0	0.00–0.00	0.00	0–1	0.00–0.00
	Internal capsule	0.00	0–1	0.00–0.00	0.00	0	0.00–0.00	0.00	0–1	0.00–0.00
	External capsule	0.00	0	0.00–0.00	0.00	0	0.00–0.00	0.00	0	0.00–0.00
	Corpus callosum	0.00	0–1	0.00–0.00	0.00	0–1	0.00–0.00	0.00	0–1	0.00–0.00
	DPWM	0.00	0–13	0.00–1.00	0.00	0–10	0.00–1.00	0.00	0–4	0.00–0.50
**CMB distribution tissue**		**median**	**range**	**IQR**	**median**	**range**	**IQR**	**median**	**range**	**IQR**
WM		5.00	0–41	1.00–12.00	5.00	0–26	0.50–9.00	1.00	0–19	0.00–7.50
GM		0.00	0–2	0.00–0.75	0.00	0–1	0.00–0.00	0.00	0–1	0.00–0.00
GM/WM junction		1.00	0–12	0.00–4.75	1.00	0–9	0.00–3.00	0.00	0–8	0.00–1.50
**CMB distribution in relation to FLAIR hyperintensity**		**median**	**range**	**IQR**	**median**	**range**	**IQR**	**median**	**range**	**IQR**
Normal FLAIR		3.50	0–29	1.00–13.75	3.00	0–17	0.50–7.50	1.00	0–18	0.00–6.50
FLAIR hyperintensity		1.00	0–14	0.00–2.00	1.00	0–9	0.00–2.00	0.00	0–10	0.00–0.00
Potential FLAIR hyperintensity		1.00	0–15	0.00–2.75	1.00	0–11	0.00–1.50	0.00	0–5	0.00–1.00
Border of FLAIR hyperintensity		0.00	0–7	0.00–1.00	0.00	0–3	0.00–0.50	0.00	0–4	0.00–0.00
**FLAIR hyperintensities volume [ml]** **(only scans with ≥ 1 CMB)**		**median**	**range**	**IQR**	**median**	**range**	**IQR**	**median**	**range**	**IQR**
		16.28	0.72–95.90	9.06–33.13	13.05	2.87–87.87	8.84–23.55	0.99	0.00–20.60	0.11–3.83
		**Whole cerebrum***N***=** **51**			**Ipsilateral cerebrum***N* **=** **47**			**Contralateral cerebrum***N***=** **47**		
**FLAIR hyperintensities volume [ml] (all scans)**		**median**	**range**	**IQR**	**median**	**range**	**IQR**	**median**	**range**	**IQR**
		19.45	0.21–96.32	9.01–34.81	16.22	0.70–87.87	8.84–31.43	0.98	0.00–43.41	0.12–4.05

⁎ - additionally distinguished (not included in MARS scale); CMB – cerebral microbleed; IQR – interquartile range; MARS – Microbleed Anatomical Rating Scale; DPWM – deep and periventricular white matter; WM – white matter; GM – grey matter; FLAIR – fluid-attenuated inversion recovery.

### 3.2 FLAIR hyperintensities

The volume of FLAIR hyperintensities in the whole cerebrum strongly correlated with the RT dose delivered to the cerebrum (*N* = 51, *r* = 0.746, *P* < 0.001) (Fig. 3), but did not correlate with time between RT completion and the MRI scan (Fig. 2), age at the time of RT, age at the time of the scan, nor with the number of CMBs. Furthermore, the volume of FLAIR hyperintensities was not statistically significantly correlated with chemotherapy, surgeries, hypertension, diabetes or smoking.

Similarly, the volume of FLAIR hyperintensities in the ipsilateral and contralateral cerebrum correlated only with the RT dose delivered to the corresponding side (*N* = 47, *r* = 0.609, *P* < 0.001; *N* = 47, *r* = 0.612, *P* < 0.001; respectively), except for a weak correlation between the volume of FLAIR hyperintensities and the number of CMBs in the contralateral cerebrum (*N* = 47; *r* = 0.339, *P* = 0.020).

Regarding the scans with ≥1 CMBs present, the volume of FLAIR hyperintensities correlated with the number of CMBs in whole, ipsilateral and contralateral cerebrum (*N* = 28, *r* = 0.404, *P* = 0.033; *N* = 25, *r* = 0.417, *P* = 0.038; *N* = 25, *r* = 0.707, *P* < 0.001; respectively).

FLAIR hyperintensities were present on all *N* = 51 scans, with a median volume of 19.45 ml (IQR = 9.01–34.84) for the whole cerebrum, 16.22 ml (IQR = 8.84–31.43) for the ipsilateral cerebrum, and 0.98 ml (IQR = 0.12–4.05) for the contralateral cerebrum. The volume of FLAIR hyperintensities in the ipsilateral cerebrum was significantly higher than in the contralateral cerebrum (*Z* = 5.97, *P* < 0.001).

## 4 Discussion

This study investigates the spatial distribution of CMBs and the degree of colocation of CMBs and FLAIR hyperintensities on follow-up MRI scans of patients who underwent RT for LGG in adulthood.

We showed that the majority of CMBs were localized in the lobar area and in deep and DPWM – and generally in WM. Only few CMBs were found in GM. Furthermore, we showed that in scans obtained up to 7 years after RT completion the majority of CMBs were not co-localized with FLAIR hyperintensities. Both may indicate that, although these two radiological markers may be of (micro)vascular origin, they also differ in several aspects and have different clinical value in the context of serving as imaging biomarkers for cognitive changes in patients with LGG treated with radiation.

Considering the hypothesis about the common origin of different radiological markers of RIBD [5] and the reported tendencies for CMBs and WM lesions (FLAIR hyperintensities) to occur in the areas with the highest delivered RT dose [14], it would be expected that CMBs and FLAIR hyperintensities are co-located (spatially overlap) or that CMBs occur on the border of FLAIR hyperintensities. However, in our population, the median number of CMBs was higher in the otherwise normal-appearing cerebral tissue on FLAIR images, rather than in the areas with FLAIR hyperintensities on the scans acquired up to 7 years since RT completion.

Only on the scans acquired 9 years after completion of RT, the mean percentage of CMBs occurring within FLAIR hyperintensities was predominant, but solely in the ipsilateral cerebrum with respect to the tumour (Fig. 4 and Supplementary Fig. 1). Furthermore, FLAIR hyperintensities were more prevalent than CMBs and occurred even on the scans where CMBs were not detected and thus we did not observe a correlation between these two types of radiological imaging biomarkers in our specific patient population. This leads us to hypothesize that although the development of CMBs and FLAIR hyperintensities could be triggered by the same mechanisms, particularly by initial endothelial damage which is then further escalated by a cascade of processes involving neuroinflammation and demyelination, the predilection for the type/diameter of the affected vessels may be different in case of FLAIR hyperintensities and CMBs.

Our findings are in contrast with a previous study by Lupo et al. in 2012 using 7 T MRI [16], where CMBs occurred mainly within the areas of WM hyperintensities, especially in the first 2.5 years after RT completion. The percentage of CMBs occurring outside WM hyperintensities in that study became dominant readily 3 years from the time of RT completion. The difference to our study may be a result of population differences, but most likely it results from the different magnetic field strength (7 T vs. 1.5 T in our study), and resulting difference in sensitivity. 1.5 T scans generally visualize a lower percentage of CMBs in comparison with 3 T and 7 T scanners, which have a higher sensitivity to observe iron deposits [21,22].

However, 7 T is still only rarely used in routine clinical practice at the moment, as opposed to 1.5 and 3 T scanners, thus limiting the direct clinical relevance of the findings at 7 T. Also, in the context of recent developments of MR-linear accelerator devices (operating at 1.5 T and below), lower field strengths than 7 T are more relevant for clinical tumour workflows in the immediate future.

Our study results are in line with the study by Bompaire et al. [15] employing T2* weighted and FLAIR MRI at 3 T in a patient population with mixed pathology (*N* = 40, glioma grade 2–4, metastasis and several others) with persistent cognitive complaints following RT. Like our study, the study by Bompaire did not observe a correlation between FLAIR hyperintensities and number and location of CMBs, which further strengthens the confidence in our results in a specific population with LGG. Furthermore, similar to studies on 7 T MRI scanners, we found a correlation between the time from RT and the number of CMBs and no correlation with the delivered RT dose [23]. However, in our study, CMBs occurred mainly on the scans acquired over 20 months since RT completion, thus later than observed on higher field scanners with the aforementioned higher iron-sensitivity, probably because the smaller CMBs were below the sensitivity threshold. On the other hand, FLAIR hyperintensities were present on all scans and did not correlate with the time from RT, but conversely did correlate with the RT dose delivered.

The findings in the current study indicate that FLAIR hyperintensities and especially CMBs spatial and temporal occurrence are still poorly defined, with CMBs being apparently (very) late imaging biomarkers of microvascular injury, making them as-such not suitable for use in the context of adaptive RT based on MRI, as well as generally in the follow-up of LGG patients. – which requires a future longitudinal study setup to elucidate the temporal occurrence in more detail, with multiple scans obtained within the same patient over time.

In this study we used the existing MARS scale, for standardized scoring of CMBs, and supplemented this scale with areas relevant for cognition that are not currently part of this scale but relevant for the purpose of this study. Using this extended MARS scale the highest median number of CMBs in our study was found in the lobar area, as reported in previous studies [8,9], and lower in the deep area of cerebrum. Furthermore, in our study most CMBs were observed in the frontal and parietal lobe, while the majority of the tumours were located in the frontal and temporal lobe. In our study CMBs were observed in the hippocampus, amygdala and cortex. Therefore, including these areas in microbleed rating scales that are used in the context of LGG would seem relevant for future studies.

Obvious limitations of this study are the relatively small study population (although not having a patient population with mixed pathology, unlike many of the other aforementioned studies), retrospective nature of the study with differing time between RT and the scan, diversity regarding cofounding factors such as chemotherapy, chemoradiotherapy, number of surgeries, different MRI protocols and scanners, different RT protocols and varying tumour location. This poses the need for future prospective studies utilizing larger populations, including follow-up scans and consistent MRI and RT protocols, and less diverse confounding factors in order to confirm and expand on the findings of this study.

A technical limitation, in the absence of an available (semi)automatic method for outlining CMBs, is the manual outlining we performed – as the manual approach could potentially depend on the observer performance and thus impact the accuracy. However, we aimed to minimize such impact and enhance the accuracy by using two observers and employed a third observer in case of discrepancies in the number of CMBs. Also, we used a modern clinical-grade semi-automatic method and approach for segmentation of FLAIR hyperintensities for the same reasons.

In conclusion, CMBs and FLAIR hyperintensities appear to be separate imaging biomarkers for radiation therapy induced microvascular damage, as they are not colocalized in patients with LGG, especially not early on after completion of RT. Our findings emphasize that in clinical practise the used magnetic field strength and the amount of time that has passed between the completion of RT and the MRI scan are both important factors to take into account while assessing either the presence or lack of CMBs. In our study population, CMBs appear to be a late marker of radiation induced microvascular damage (predominantly over 20 months after RT), highlighting the need for additional assessment of earlier markers such as FLAIR hyperintensities and subsequent joint interpretation of these markers in the context of the dose delivered to the tissue.

## Ethics statement

This study is based on retrospective data of patients with lower grade glioma (LGG).

This study was approved by the institutional review board and the need for written informed consent was waived (because of the retrospective nature of the study).

## Patient consent statement

This manuscript does not include case details or other personal information or images of patients that contain identifiable information and therefore no patient consent is required per Elsevier's Patient Consent Guidelines (https://www.elsevier.com/about/policies/patient-consent).

## CRediT authorship contribution statement

**Justyna Kłos:** Conceptualization, Data curation, Formal analysis, Investigation, Methodology, Project administration, Validation, Visualization, Writing – original draft, Writing – review & editing. **Reina W. Kloet:** Conceptualization, Formal analysis, Validation, Writing – review & editing. **Hiska L. van der Weide:** Data curation, Resources, Software, Validation, Writing – review & editing. **Kelvin Ng Wei Siang:** Data curation, Resources, Software, Validation, Writing – review & editing. **Peter F. Sinnige:** Data curation, Resources, Software, Validation, Writing – review & editing. **Miranda C.A. Kramer:** Data curation, Resources, Software, Validation, Writing – review & editing. **Rudi A.J.O. Dierckx:** Conceptualization, Funding acquisition, Investigation, Methodology, Project administration, Supervision, Validation, Writing – review & editing. **Ronald J.H. Borra:** Conceptualization, Resources, Software, Validation, Writing – review & editing. **Anouk van der Hoorn:** Conceptualization, Formal analysis, Investigation, Methodology, Project administration, Supervision, Validation, Visualization, Writing – review & editing.

## Declaration of Competing Interests

The authors declare that they have no known competing financial interests or personal relationships that could have appeared to influence the work reported in this paper.

## Appendix Supplementary materials

Image, application 1

Image, application 2

## Footnotes

Supplementary material associated with this article can be found in the online version at doi:10.1016/j.redii.2023.100033.
