# Supplementary material for: Spatial distribution of cerebral microbleeds and FLAIR hyperintensities on follow-up MRI after radiotherapy for lower grade glioma
Source: Res Diagn Interv Imaging. 2023 Aug 14;7:100033. doi: 10.1016/j.redii.2023.100033 (PMC11265380; doi:10.1016/j.redii.2023.100033)
Supplement: Supplementary file 1 [file mmc1.pdf]

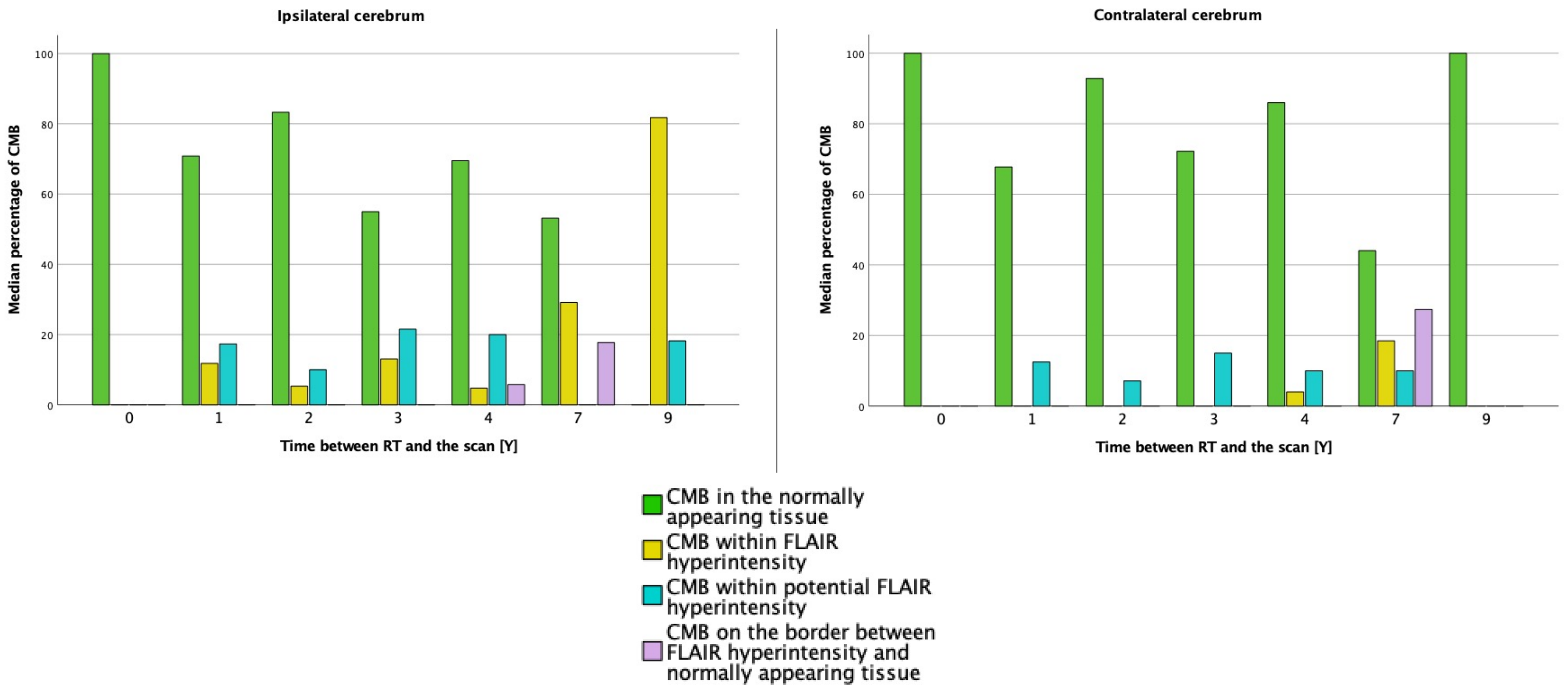

Ipsilateral cerebrum
Contralateral cerebrum
Median percentage of CMB
100
80
60
40
20
0
0
1
2
3
4
7
9
Time between RT and the scan [Y]
CMB in the normally appearing tissue
CMB within FLAIR hyperintensity
CMB within potential FLAIR hyperintensity
CMB on the border between FLAIR hyperintensity and normally appearing tissue
