# Supplementary material for: Spatial distribution of cerebral microbleeds and FLAIR hyperintensities on follow-up MRI after radiotherapy for lower grade glioma
Source: Res Diagn Interv Imaging. 2023 Aug 14;7:100033. doi: 10.1016/j.redii.2023.100033 (PMC11265380; doi:10.1016/j.redii.2023.100033)
Supplement: Supplementary file 2 [file mmc2.docx]

**SUPPLEMENTARY FIGURE 1 Caption:**

Median percentage of different types of CMBs in ipsilateral and contralateral cerebrum (n=25) depending on the time between RT completion and the scan (in years). Both sides display predominance of CMBs occurring in the normally appearing tissue on FLAIR images. Only in the ipsilateral hemisphere the median percentage of CMBs within FLAIR hyperintensities became dominant on the scans acquired 9 years after completion of RT.
